# Supplementary material for: Leptin-activated hypothalamic BNC2 neurons acutely suppress food intake
Source: Nature. 2024 Oct 30;636(8041):198–205. doi: 10.1038/s41586-024-08108-2 (PMC11618066; doi:10.1038/s41586-024-08108-2)
Supplement: Supplementary file 1 — Reporting Summary [file 41586_2024_8108_MOESM1_ESM.pdf]

nature portfolio

Corresponding author(s): Jeffrey M. Friedman

Last updated by author(s): Aug 15, 2024

Reporting Summary

Nature Portfolio wishes to improve the reproducibility of the work that we publish. This form provides structure for consistency and transparency in reporting. For further information on Nature Portfolio policies, see our Editorial Policies and the Editorial Policy Checklist.

Statistics

For all statistical analyses, confirm that the following items are present in the figure legend, table legend, main text, or Methods section.

n/a	Confirmed	
☐	☒	The exact sample size (*n*) for each experimental group/condition, given as a discrete number and unit of measurement
☒	☐	A statement on whether measurements were taken from distinct samples or whether the same sample was measured repeatedly
☐	☒	The statistical test(s) used AND whether they are one- or two-sided<br>*Only common tests should be described solely by name; describe more complex techniques in the Methods section.*
☒	☐	A description of all covariates tested
☐	☒	A description of any assumptions or corrections, such as tests of normality and adjustment for multiple comparisons
☐	☒	A full description of the statistical parameters including central tendency (e.g. means) or other basic estimates (e.g. regression coefficient) AND variation (e.g. standard deviation) or associated estimates of uncertainty (e.g. confidence intervals)
☐	☒	For null hypothesis testing, the test statistic (e.g. *F*, *t*, *r*) with confidence intervals, effect sizes, degrees of freedom and *P* value noted<br>*Give P values as exact values whenever suitable.*
☒	☐	For Bayesian analysis, information on the choice of priors and Markov chain Monte Carlo settings
☒	☐	For hierarchical and complex designs, identification of the appropriate level for tests and full reporting of outcomes
☒	☐	Estimates of effect sizes (e.g. Cohen's *d*, Pearson's *r*), indicating how they were calculated

Our web collection on statistics for biologists contains articles on many of the points above.

Software and code

Policy information about availability of computer code

Data collection	Real-time place preference assay data were collected and analyzed using Ethovision XT13. Fiber photometry data were collected through the Fiber Photometry system from Tucker-Davis Technologies (RZ5P, Synapse)
Data analysis	The snRNA-seq data were analyzed using Cell Ranger version 5.0, Seurat v4 (v4.0.3), and R studio (2022.07.2). Matalab 2022b was used for the analysis of fiber photometry data. The analytic code is available at this GitHub repository: https://github.com/yuqiyuqitan/BNC2.

For manuscripts utilizing custom algorithms or software that are central to the research but not yet described in published literature, software must be made available to editors and reviewers. We strongly encourage code deposition in a community repository (e.g. GitHub). See the Nature Portfolio guidelines for submitting code & software for further information.

Data

Policy information about availability of data

All manuscripts must include a data availability statement. This statement should provide the following information, where applicable:

- Accession codes, unique identifiers, or web links for publicly available datasets
- A description of any restrictions on data availability
- For clinical datasets or third party data, please ensure that the statement adheres to our policy

All data generated or analyzed during this study are included in the manuscript and supporting files. The raw sequencing data have been deposited in the Gene Expression Omnibus and are available under the accession number GSE249564.

Research involving human participants, their data, or biological material

Policy information about studies with human participants or human data. See also policy information about sex, gender (identity/presentation), and sexual orientation and race, ethnicity and racism.

Reporting on sex and gender	NA
Reporting on race, ethnicity, or other socially relevant groupings	NA
Population characteristics	NA
Recruitment	NA
Ethics oversight	NA

Note that full information on the approval of the study protocol must also be provided in the manuscript.

Field-specific reporting

Please select the one below that is the best fit for your research. If you are not sure, read the appropriate sections before making your selection.

☒ Life sciences ☐ Behavioural & social sciences ☐ Ecological, evolutionary & environmental sciences

For a reference copy of the document with all sections, see nature.com/documents/nr-reporting-summary-flat.pdf

Life sciences study design

All studies must disclose on these points even when the disclosure is negative.

Sample size	The sample size were determined based on similar studies in the filed (Xu et al., Nature. 2018. PMID: 29670283; Biglari et al., Nature Neuroscience 2021. PMID: 34002087; Chen et al., Cell.2015. PMID: 25703096; Aponte et al., Nature Neuroscience 2011. PMID: 21209617.).
Data exclusions	No data were excluded.
Replication	Experiments were performed independently across multiple animal replicates, as detailed in the paper.
Randomization	Mice were randomly assigned to control and experimental groups and littermates were used when possible.
Blinding	Behavioral data were collected and analyzed blind to whether animals were in control or experimental group.

Reporting for specific materials, systems and methods

We require information from authors about some types of materials, experimental systems and methods used in many studies. Here, indicate whether each material, system or method listed is relevant to your study. If you are not sure if a list item applies to your research, read the appropriate section before selecting a response.

Materials & experimental systems

n/a	Involved in the study	
☐	☒	Antibodies
☒	☐	Eukaryotic cell lines
☒	☐	Palaeontology and archaeology
☐	☒	Animals and other organisms
☒	☐	Clinical data
☒	☐	Dual use research of concern
☒	☐	Plants

Methods

n/a	Involved in the study	
☒	☐	ChIP-seq
☒	☐	Flow cytometry
☒	☐	MRI-based neuroimaging

Antibodies

Antibodies used	cFos antibody (1:1000, Synaptic systems, #226308), pSTAT3 antibody (1: 1000, Cell Signaling Technology, #9145s), GFP (1:1000, abcam, ab13970), RFP (1:1000, Rockland, #600-401-379), anti-NeuN Alexa Fluor 647-conjugated antibody (Abcam ab190565) (0.5 µl per million nuclei), and TotalSeq anti-Nuclear Pore Complex Proteins Hashtag antibody (BioLegend 682205) (0.5mg per million

	nuclei).
Validation	cFOS antibody (PMID: 31257028)<br>pSTAT3 antibody (PMID: 32699414)<br>GFP antibody (PMID:36413988)<br>RFP antibody (PMID: 35705049, 35659861)<br>NeuN antibody (PMID: 36071158, 12556490)<br>Nuclear pore complex (PMID: 12556490, 11448990)

Animals and other research organisms

Policy information about studies involving animals; ARRIVE guidelines recommended for reporting animal research, and Sex and Gender in Research

Laboratory animals	Adult male and female (8-20 weeks) mice from strains: C57BL/6J (wild type; number 000664, The Jackson Laboratory), NPY-IRES2-FlpO-D (The Jackson Laboratory, stock no. 030211), Rosa26-LSL-Cas9 (The Jackson Laboratory, stock no. 024857), BNC2-P2A-iCre (made in-house).
Wild animals	This study did not involve wild animals
Reporting on sex	Some experiments were performed using both male and female mice and some were performed using male mice only. The sex information has been stated in the manuscript.
Field-collected samples	This study did not involve samples collected in the filed.
Ethics oversight	All animal experiments were approved by the Institutional Animal Care and Use Committee at Rockefeller University and were carried out in accordance with the National Institutes of Health guidelines.

Note that full information on the approval of the study protocol must also be provided in the manuscript.

Plants

Seed stocks	NA
Novel plant genotypes	NA
Authentication	NA
